# Advances in AI-based diagnosis of Alzheimer’s disease using MRI: a comprehensive survey

**DOI:** 10.3389/fmed.2026.1767090

**Published:** 2026-06-01

**Authors:** Hasan Issa Raheem Alyaqoobi, Jose Manuel Lopez-Guede, Omer Asghar Dara, Jose Antonio Ramos-Hernanz, Iñigo Aramendia, Daniel Teso-Fz-Betoño

**Affiliations:** 1 Department of Systems Engineering and Automatic Control, Faculty of Engineering of Vitoria-Gasteiz, University of the Basque Country (UPV/EHU), Vitoria-Gasteiz, Spain; 2 Department of Electric Engineering, Faculty of Engineering of Vitoria-Gasteiz, University of the Basque Country (UPV/EHU), Vitoria-Gasteiz, Spain

**Keywords:** AI in radiology, Alzheimer’s disease, cognitive disorder classification, deep learning in medical imaging, neurodegenerative disease diagnosis

## Abstract

Artificial intelligence (AI), especially Deep Learning (DL), has been shown significant in accelerating the detection and diagnosis of neurological disorders via medical imaging. This study is mainly focused on Alzheimer’s disease (AD), which reveals distinctive structural modifications observable by Magnetic Resonance Imaging (MRI). Although several studies employing convolutional neural networks (CNNs) and other artificial intelligence models indicate promising diagnostic accuracy, many issues related to methodology exist. This research offers a comprehensive assessment of recent studies (2000–2025) to synthesize the key limitations limiting the clinical application of AI for AD detection using MRI. The study identify the main challenges, namely: (1) restricted access to extensive, curated, and diverse multimodal datasets; (2) elevated model complexity with associated risks of overfitting on small cohorts; (3) insufficient interpretability and clinical validation of AI decisions; (4) computational inefficiency and excessive energy consumption; and (5) challenges in generalizing models across heterogeneous cohorts and imaging guidelines. Our study indicates that modern research frequently emphasizes marginal improvements in accuracy rather than solving these essential translational obstacles. The authors conclude by outlining essential research progressions, highlighting the necessity for federated learning for dealing with data scarcity, the advancement of explainable AI (XAI) frameworks, and the creation of standardized benchmarking protocols to flexible, clinically-adoptable AI methods for early AD detection.

## 1 Introduction

AD is a neurodegenerative disorder marked by progressive cognitive decline, primarily defined by what is called dementia and impairments with memory functions in the brain (1). The main objective that leads as to this study were risks and limitations such as (1) many datasets limits generalizability (2) the disease complexity as the progress varies among patients in which AI tools might find difficulties in some cases. (3) Disease interpretation as doctors needs some training to interpret or understand AI tools’ explanations. (4) The efficiency and hardware resources required for such ML/DL models. In 2010, approximately 35 million individuals worldwide were affected, with projections indicating to reach 85 individuals by 2050 (2). For instance, approximately five million individuals were diagnosed with AD in the United States of America in 2010, incurring an estimated annual waste of money of approximately $230 million for the providing of treatment, medication, and maintenance (3). Only in Europe, more than 6.9 million people are affected by the disease, and the authors are still counting as this number is expected by health organizations to almost be double by the year 2050. AD progresses gradually, first impairing new or recent memory before affecting middle or long term things (4). This gradual and unexpected progress of AD will be imperative to accurately and promptly diagnose or even treatment of the structural regions in the brain that happened during the initial spread of the damage which is considered beneficial for a point of preventative measures (5). It is extremely well known among researchers in this field that the tracking of AD may take several years before the appearance of clinical symptoms, with an estimated average duration of more than ten years (6). Considering this slow development period preceding the clinical signs, researchers are really struggling to identify the best strategies for the early detection of the disease in individuals with high probability of being suspectable (7). In case of MRI, which is a type of scan that uses magnetic fields and radio waves, it identifies alterations in the dimensions of specific brain regions (8). The use of dimensions to assess the areas experiencing atrophy through the advancement of the disease can be used as a diagnostic indicator for early diagnosis.

**FIGURE 1:**
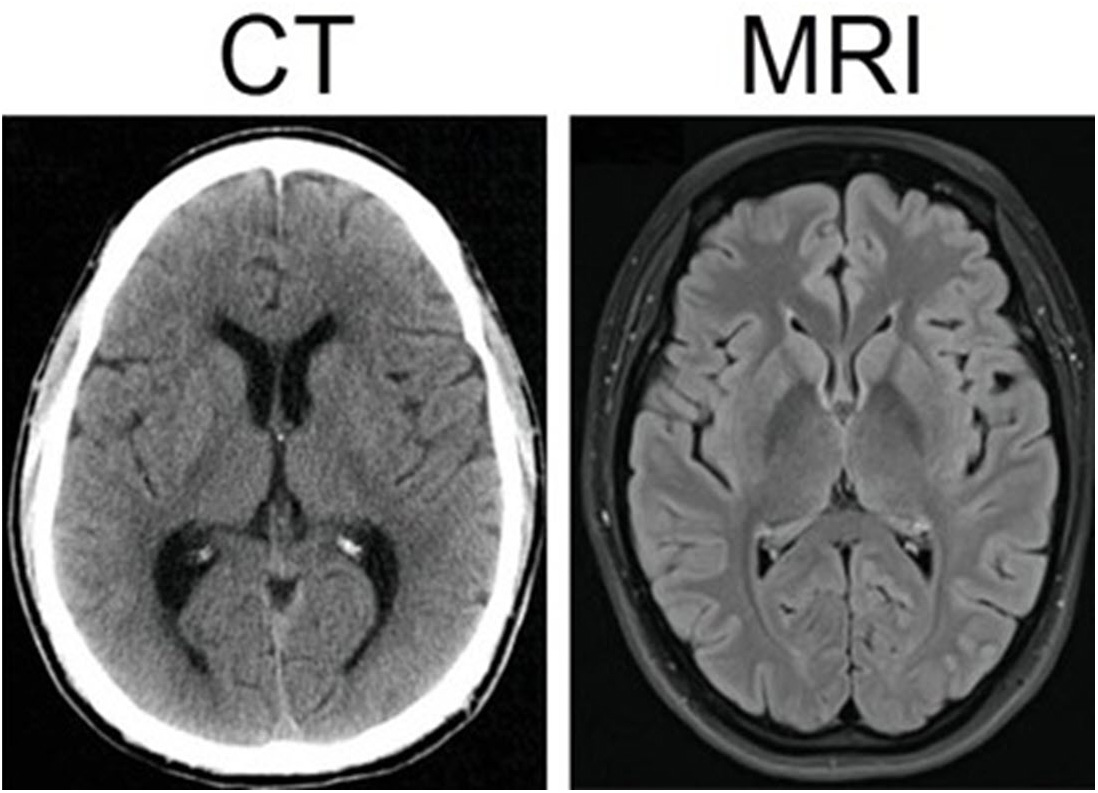
Structural representation of brain in normal and AD cases.

AD has a prevalence rate of two to five percent among the elderly population, and in some cases, it can also manifest in younger individuals (9) (Epidemiology of Aging and Associated Cognitive Disorders: Prevalence and Incidence of Alzheimer’s Disease and Other Dementias 2019). There is a hypothesis that it is related to cellular impairment within the hippocampus, a brain region known for its production of acetylcholine, and to AD (10). Brain cells, or neurons, that have been impaired undergo the accumulation of plaques, leading to a substantial loss of cellular population (11). The impaired region of the cerebral cortex and the neurotransmitter acetylcholine play a crucial role in memory formation (12). Besides, a noticeable manifestation of AD is the inability to recognize novel information, such as the recollection of a recently acquired address, along with challenges in maintaining proper orientation (13). However, memories of events that occurred in the past tend to exhibit less impairment (14). Dementia among the old ages is the primary reason for AD (15), as recent research proposes that these types of diseases will experience an increase within the next 22 years (16). While dementia is normally diagnosed in the clinic as physicians would read history and do the examination, imaging techniques in scope of computer science are very important for evaluating or assessing patients to have dementia (17). These techniques help to exclude potential other causes of dementia and identify certain factors contributing to patient’s condition (18). MRI and Computed Tomography (CT) scanning have the potential to identify non-degenerative causes of dementia that may be easy to be treated or can be healed (19). However, as declared by neurological doctors and experts, the usage of CT scans and MRIs scans does not always play a real role in dementia illness (20). Cerebral atrophy can be visualized through X-ray imaging in specific images or instances (21). A typical individual without any cognitive impairments should show a healthy brain scanned image, whereas those with cognitive impairments may show different patterns when compared with the above-mentioned standard scans (22). Current investigations are currently focused on the advancements of image manipulation techniques for the early detection of AD during its early stage, potentially even before the symptoms that can be considered as AD (23). Several recent articles were proposed in order to collect relevant and prior knowledge that focus on the main spots of CT and MRI scans in the human brain in all sides (24). The application of AI here is by using Machine Learning (ML) and DL approaches in disease diagnosis by using MRI or CT of the brain as a step for creating the AI learning model (25). This approach can be employed to diagnose diseases associated with the nervous system, including AD (26). This section shows several studies, encompassing research on brain imaging techniques, Otsu thresholding, and diagnosing AD. The principle of the formation of images is the utilization of MRI within the brain to spot the affected regions (27). Figure 1 illustrates the effect of AD on the brain and how it causes shrinkage of the cerebral cortex and hippocampus (28).

This survey examines the use of artificial intelligence models based on Magnetic Resonance Imaging (MRI) data to diagnose AD automatically with the best results (29). It explicitly considers how memory relies on structural organization and can be interpreted using AI techniques (30). AD is identified by the disruption of the structure of the brain caused by the degeneration and eventual death of neurons (31). As previously stated, the disturbance has a negative impact on the processes of information retrieval and memory retention (32). Parkinson disease (PD) is primarily related to dopamine disruption, whereas AD is recognized by hippocampus and cortical atrophy shown using MRI. By focusing on MRI biomarkers rather than using neurotransmitter imaging. AD does not exhibit a unique brain region or distinct impact that directly corresponds to cognitive impairment (33). However, memory of the brain is intricately connected to the cognitive and neurological processes inside the brain and can be affected when there are mental or psychological changes in human behavior (34). The worldwide appearance of this disease is experiencing a significant upward trend through the following years. This disease is remarked by a visible and steady reduction in cognitive function while showing issues in human movement, thinking, and speaking. Dementia shows a wide range of problems caused by brain damage or disease, resulting in terrible impacts on memory, cognition, and behavior (35). The adjustments significantly impact the daily duties conducted by humans (36). An overview of recent studies relating AD to artificial intelligence and its limitations is presented in this review. This study aims to explore various techniques that can enhance the accuracy of the diagnosis of AD using features extracted from MRI images. This survey considered some parameters such as model constraints, hardware requirements, and the energy consumption associated with processing power and model complexity. These approaches are inadequate, given the intricate nature of the disease. Pietrzak’s in 2018 assessment examined nuclear medicine imaging devices but did not address any potential future developments in artificial intelligence (37). In 2016, Alberdi’s analysis primarily examined multimodal signals and noise classification in scans (38). However, the paper did not discuss the potential application of ML techniques to analyze these pictures, although it highlights the importance of early disease identification. In a study conducted by (39), it was claimed that Resnet 50 with an Adaptive Rider Optimization outperformed other ML algorithms without considering other techniques. In 2025, Gami et al. (40) reviewed AD disease and highlighted the role of advanced ML models were commonly employed in assessing disease severity. The reason for this is that such approaches as ANN, unlike SVM, are prone to limited local drawbacks, and DL methods or hybrid models have the potential to yield satisfactory results (41).

The structure of the paper is as follows. Section 2 shows the review methodology and strategies utilized while searching the articles related to detecting AD through the analysis of associated images, including artificial intelligence approaches such as DL methods. Section 3 explains the observed changes when using CT and MRIs. Section 4 shows the literature review of the recent research studies regarding the diagnosis of AD and defects in the brain using AI techniques. Section 5 presents a discussion expressing an impartial viewpoint on upcoming and promising trends and other relevant factors. The last section provides the key concepts in the field of AD and artificial intelligence presented throughout the study and the final evaluation of the topic.

## 2 Review methodology

**FIGURE 2:**
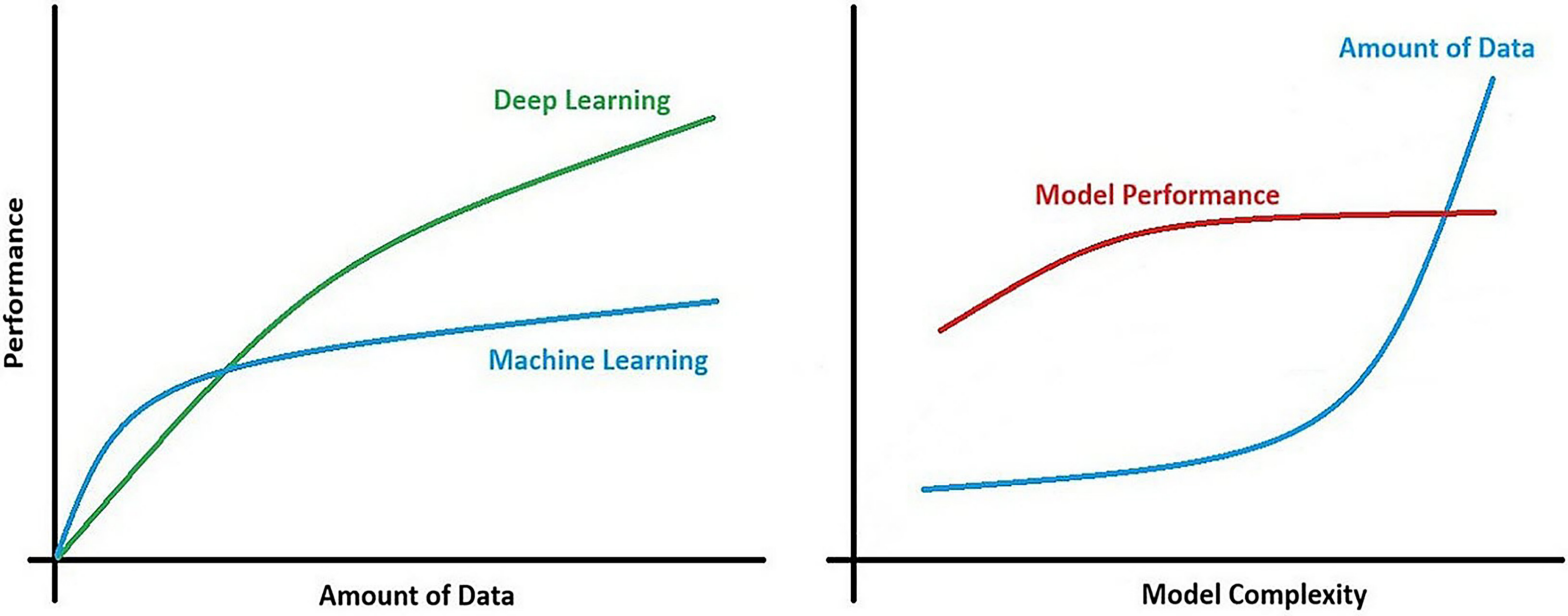
Percentage of each scholarly database used for this survey.

**FIGURE 3:**
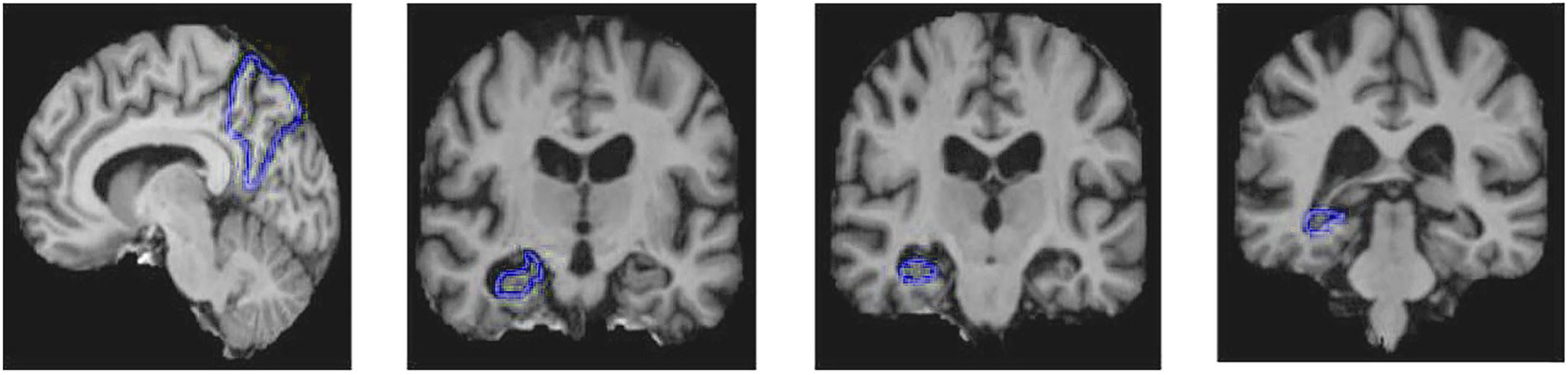
Percentage of applying ML and DL Approaches.

The study examined many contributions related to the detection of AD using AI technologies from the year 2000. This examination focused on prominent scholarly databases, including Web of Science (WoS), PubMed Central, MEDLINE, SpringerLink, ScienceDirect, and MDPI. The search was implemented by using many scientific words, such as “Alzheimer’s Severity,” “Artificial Intelligence in Alzheimer’s,” and “Deep learning in the diagnosis of brain issues,” and checking the study articles and proceedings related to the review. Figure 2 shows the percentage of scholarly databases utilized, and Figure 3 shows the percentages for the use of ML (36%) and DL (64%) approaches in the field.

**FIGURE 4:**
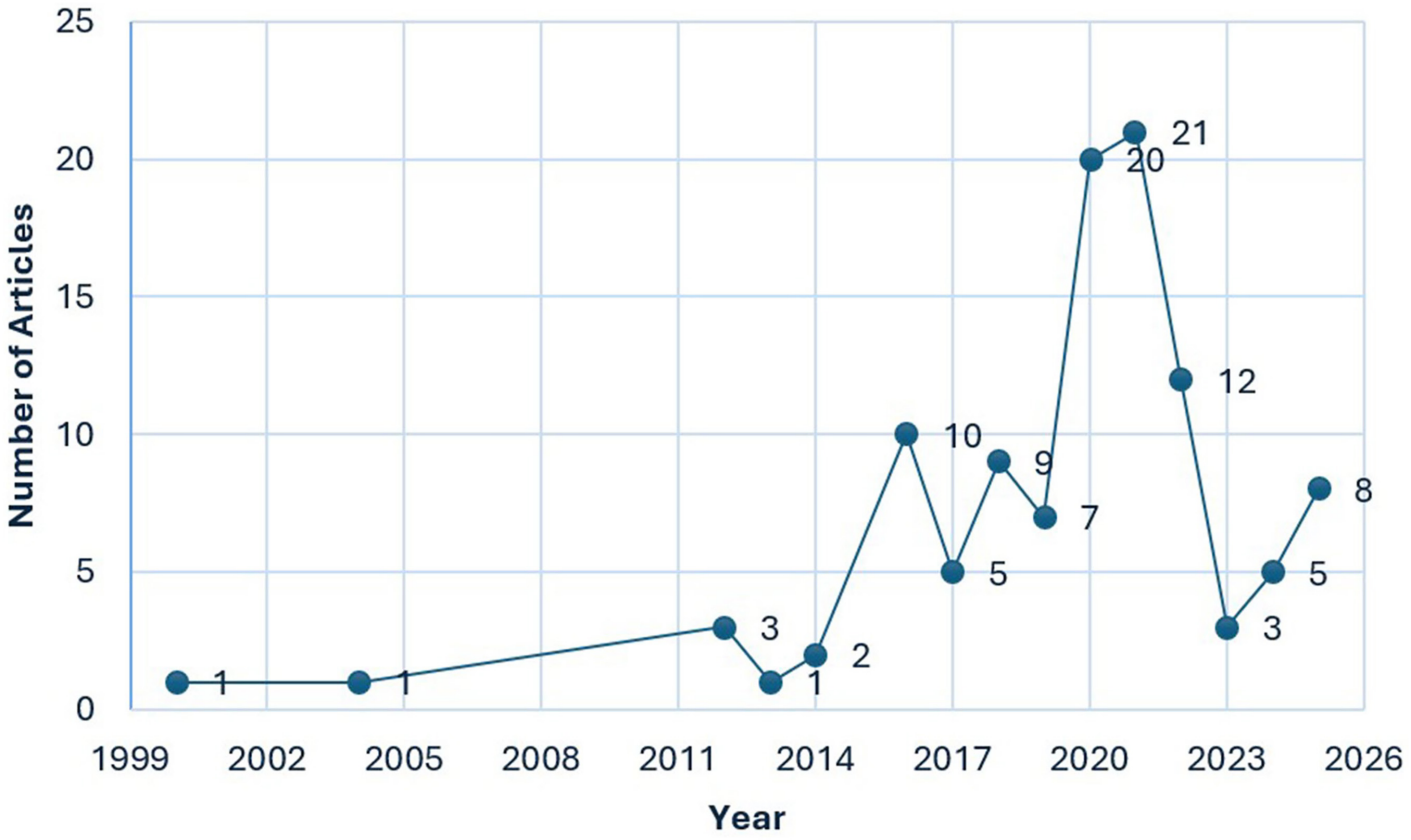
Review of indexed publications per year.

The review methodology’s findings were validated using high-ranking scientific indexes, such as the Web of Science and PubMed Databases. The study used these indexes to ensure the findings’ inclusiveness and identify related studies. It was selected based on the applications of the ML and DL approaches in biomedical studies, focusing on the stages of the disease’s progression, including “exposed,” “subclinical,” “clinical,” and “recovery.” The search lists covered a substantial part of the existing literature on the applied approaches utilized to diagnose AD in its different stages. Figure 4 displays the quantities of each utilized indexed database or publisher associated with the year of publication.

**FIGURE 5:**
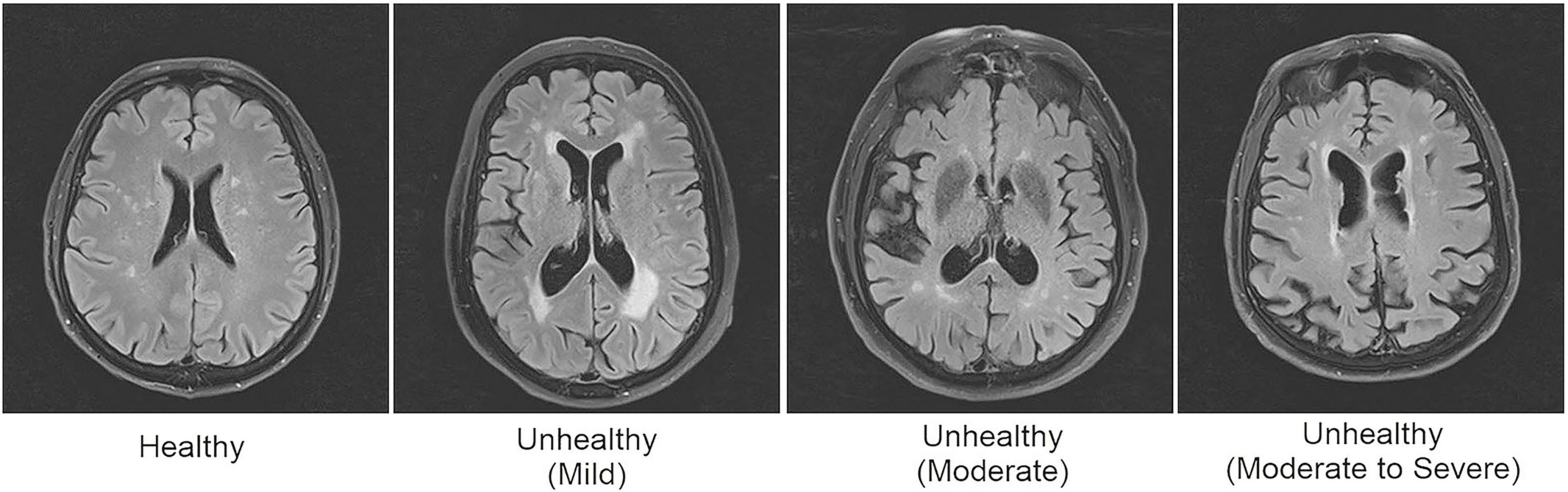
PRISMA flowchart diagram of our study.

In addition, the authors utilized ResearchGate and Google Scholar platforms for more article searching. This methodology necessitated examining the references cited in the publications to improve our search and find any neglected articles. In order to show how the studies were identified, screened, and checked to be used in our systematic review, the study added PRISMA flowchart (42). Figure 5 shows the process of study selection articles using PRISMA. This research proceeded until September 30, 2025.

## 3 CT vs. MRI imaging in AD

### 3.1 Observed changes using a CT

CT scans are frequently used for diagnostic purposes and to investigate the correlation between brain function and behavior (43). However, their primary application arises when MRI is contraindicated, as MRI is presently the preferred modality for assessing neurodegenerative disorders (44). As an individual becomes older, cellular degeneration occurs in the brain concurrently with other organs. MRI and CT scans are well known as data for medical imaging to create visual aspects of the internals of the human body (45). These imaging generated from these scans take part in the diagnostics and detection process that assist in treatment and assessing various medical conditions (46).

In biomedical studies, especially in brain imaging, cellular analysis is visually represented by cerebral atrophy, although it’s considered that atrophy is a term typically associated with pathological condition (47). However, the term “cerebral atrophy” has occasionally been misused to refer to the reduction in cell volume, as shown in this review (48). However, it is essential to note that cerebral atrophy is not universally observed in all individuals with dementia (49). Approximately 66% of patients diagnosed with AD exhibit cerebral atrophy using CT scans; these scans may demonstrate normal findings especially during the initial process of the physical disorder (50). From a cognitive perspective point, various regions in the brain are affected such as the temporal neocortex, parietal lobe, and the cerebral cortex resulting in overall brain atrophy (51). Additionally, the hippocampus is affected as the disease progresses from normal cognitive function to impairment and dementia (52).

Distinguishing AD from normal growing in age or other reasons of cognitive issue or impairment is impossible using a standard CT scan (53). An angled axial CT scan is employed to assess the status of the temporal lobe, with particular emphasis on its internal region. The axial CT scan can detect a reduction in the dimension of the medial section of the temporal lobe (54). This method demonstrates a high rate in sensitivity and specificity findings as an indicator of AD. The minimum acceptable threshold has to identify the observable variance among those diagnosed with AD and those in the control group who are in good health (55). Individuals without specialized training or expertise in the field of cognitive impairment, as well as non-organic factors such as depression, may result in a degree of uncertainty in distinguishing between AD and alternative forms of dementia, such as vascular dementia. However, their ability to accurately differentiate between these conditions may be limited (56).

Hippocampal evaluations are a radiological marker that has shown a retraction accuracy of 91% in the initial assessment of dementia. The elements are impacted during the initial phases of AD, accounting for the enlargement of the longitudinal fissure in individuals diagnosed with AD (57). The diagnostic process exhibits a high sensitivity, yet lacks commensurate specificity for example, “a sensitivity of 91% and a specificity of 89% as a predictor of decline” (58). One of the applications of CT scans is their frequent use in urgent medical scenarios, as they have generated fast results, and this normally assists healthcare employees to deliver diagnostic results and primary care (45).

### 3.2 Observed changes using an MRI

**FIGURE 6:**
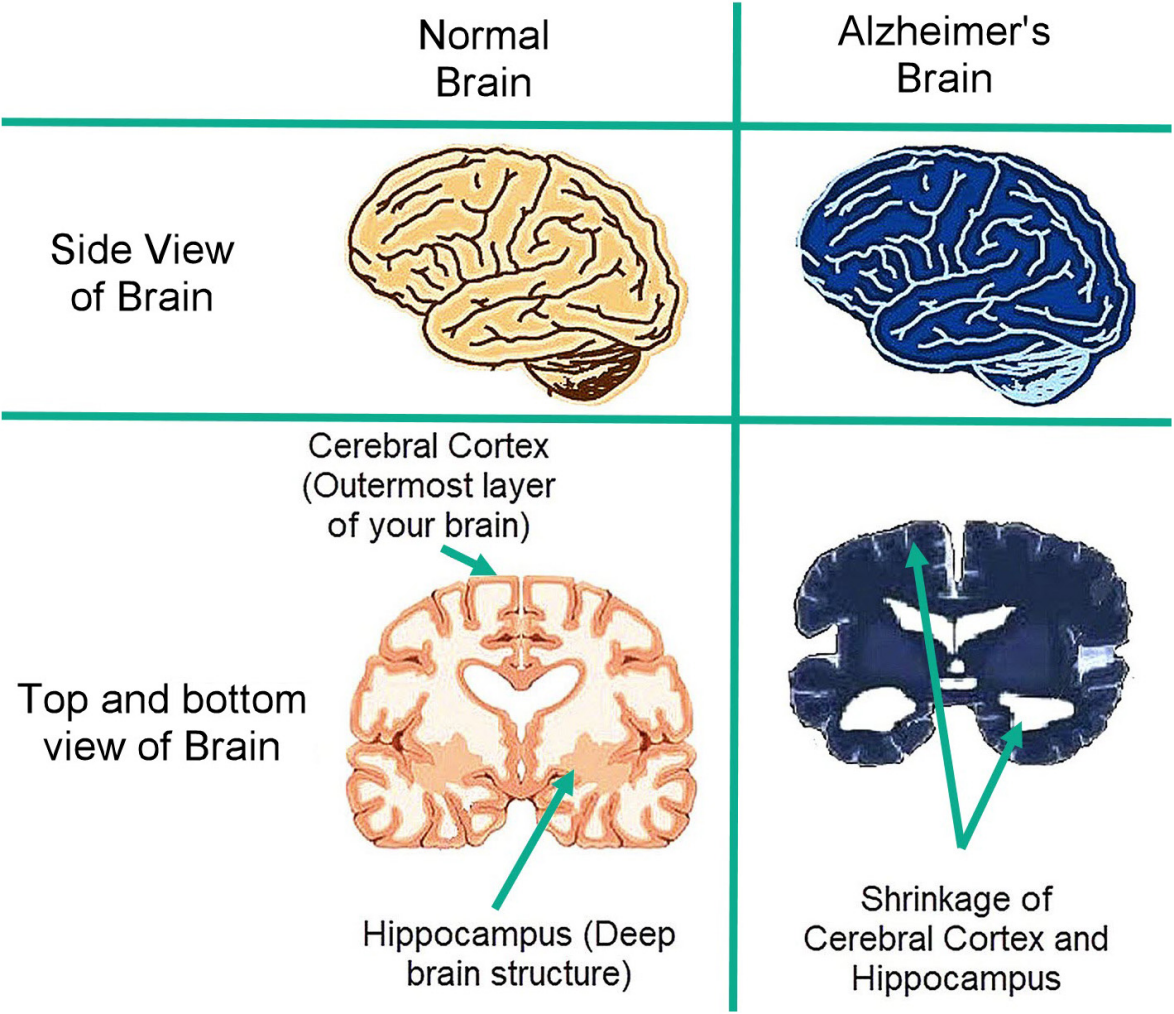
CT (left) and MRI (right) scans illustrating hippocampal atrophy in AD.

The primary distinction between these two diagnostic imaging modalities lies in their respective operation: MRI employs powerful magnetic fields to capture images, while CT scans utilize X-rays, used when MRI is not indicated or not identified (59). Individuals exhibiting cognitive impairment may be identified as early as 3 years before the manifestation of clinical symptoms, in contrast to a control group (60). The interest in evidence exists regarding the atrophy of internal structures within the temporal lobe, specifically the hippocampus and entorhinal cortex, happening early in the progression of the disease, potentially preceding the incorporation of symptoms (61). This phenomenon has been observed in clinical settings using structural MRI in individuals with Mild Cognitive Impairment (MCI). Upon the manifestation of the disease’s mild symptoms, it is observed that the volume of the hippocampus has potentially undergone a reduction exceeding 25% (62). The clinical checks through the symptoms of memory loss, patient’s report on cognitive evaluation tests, and pathological results are associated with the volume of the hippocampus. However, an alternative perspective lacks any definitive correlation between lesions observed during the progression of dementia, such as hyperexcitability lesions in white matter as seen through MRI, and the extent of cognitive impairment symptoms, even after considering the age factor (63). However, any alternative perspective or any clear evidence to the correlation between lesions observed during the progression of AD (26). Recent experiments published in the field have demonstrated an important association between hyperexcitability lesions within white matter, as clearly shown using MRI and advancement of AD. These lesions disrupt the connectivity between cortical and subcortical patterns or regions which hold communications (64). While hippocampal atrophy serves as a critical biomarker for AD, white matter hyperintensities contribute to executive dysfunction, memory deficits, and an accelerated disease trajectory. Furthermore, their existence often indicates concurrent vascular pathology, which makes it worse for cognitive decline and emphasizes the demand of utilizing the multimodal imaging techniques for early detection of these kinds of diseases (65). Table 1 shows a short comparison between MRI and CT scans, while Figure 6 shows the CT and MRI images scanned from the brain.

**TABLE 1:** Comparison between MRI and CT scans.

Property	MRI	CT
Method	Magnetic fields and radio waves	X rays
Utility	Soft tissues	Bone and dense tissues
Radiation	None	Ionizing
Time of scan	Up to 60 min	Few minutes
Cost	Expensive	Less expensive
Availability	Less available	More available

Several medical tests are conducted to identify and evaluate a person’s health, including blood, urine, and genetic tests. A blood or urine test can detect other symptoms, including vitamins and nutrients, infections, and liver, kidney, and thyroid function (66). Genetic testing is conducted to identify family patterns of dementia. As technological advances continue to progress, so too do medical procedures. The usage of brain scan is useful for the diagnostic techniques created to check or consider as a factor the identification of AD (67). These methodologies involve monitoring specific brain regions involved to the hippocampus, cerebral cortex, memory, communication, and reasoning to obtain indications. The alteration in size of these components is contingent upon the presence and advance of AD (68).

**FIGURE 7:**
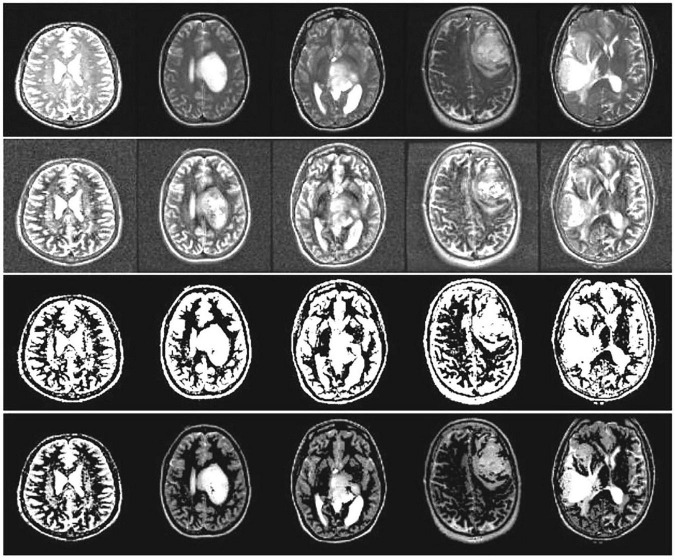
Factors that affect the occurrence of the disease of AD.

MRI-based changes in the entorhinal cortex and hippocampus are the main biomarkers for early diagnosis of AD. Many AI approaches are trained on public datasets like ADNI or OASIS are influenced by these biomarkers or “features” in the categorization of AD progression (69). The effect of antioxidants, specifically vitamin E and zinc, was examined in relation to longevity, cognitive decline, and dementia (70). (71) identified oxidative stress as a critical factor in aging and Alzheimer’s disease, emphasizing its implications for future research. A study created by (72) to characterize and quantify the population exposed occupationally to electromagnetic fields (EMF) from MRI devices, as well as to find factors influencing the likelihood and nature of referenced illness. The data depicts contemporary and historical advances in magnetic resonance imaging while showing a comprehensive characterization of the occupationally revealed demographic results. Clinical instructions should be taken into account, as there are a significant number of employees who may be put at risk by MRI-related electromagnetic fields. The nature and frequency of potential exposure are accidental upon the specific job and the type of workplace (73). The rate of development of AD is influenced by multiple factors, as depicted in Figure 7.

Various methodologies for generating medical images have been examined, along with instances of the biological applications provided (74). CBIR, also known as content-based image retrieval, encompasses methods that enable the identification of digital images based on their visual attributes (75). Computer vision techniques are encountering picture retrieval issues in large databases related to computer applications (76).

## 4 AI Approaches in literature

This section contains a literature review of the study, presented in the following manner: section 4.1 explains image processing and computer vision, as well as the ways in which these technologies enable the interpretation and learning of images. In the following section 4.2, the various techniques of ML and the limitations of their applicability are presented. A discussion of the DL approaches and how they were used is included in section 4.3. The Ensemble and Entropy methods, as well as Biological Networks, are explained in sections 4.4, 4.5, and 4.6, respectively.

### 4.1 Image processing

Computer vision and image enhancement comprise the two primary branches of image processing. Enhancing images use various techniques, such as blur filters and contrast enhancements, which aim to improve their visual quality and ensure their accurate representation in the intended output medium, such as a printer or computer monitor (77). Computer vision, on the other hand, enables the interpretation and comprehension of images, making them useful for learning and robotics (78). In its context, image processing refers to applying signal processing techniques to manipulate image inputs, such as photographs or movie scenes. The resulting output from the image processor can manifest as an image itself or a collection of distinctive symbols or variables associated with the processed image (79). From a study created by Alabduljabbar et al. (80), a novel image fusion-based threshold prototype has been proposed also to improve segmentation accuracy in AD. This study integrated multiple imaging modalities such as MRI, CT, or multiple MRI sequences into fused data, which enhances the visibility of structural changes in these patterns. The methodology of this study applied an adaptive thresholding across many intensity levels which created a multilevel segmentation of color-coded zones. Each zone meant to have a distinct tissue characteristic such as healthy tissue, atrophied regions, and lesion-affected areas. This would allow for clearer differentiation of pathological observation to the changes. Combining this grayscale data with colored enhanced structural mapping should reduce the noise and improve boundary detection. Thus, provided more reliable biomarkers for the case of applying these kinds of data to AI approaches.

Recent developments in DL research have shown that the capacity of AI approaches can detect the shared anatomical characteristics in various neurodegenerative conditions (81). Both AD and Lewy Body Dementia (LBD) frequently expose hippocampal and cortical atrophy, whereas vascular dementia is often related with white matter hyperintensities that contribute to cognitive deterioration (82). AI approaches, when trained on multimodal MRI datasets, have the ability to distinguish these differences, this will enhance the diagnostic precision rates and clinical utility. These AI approaches emphasize the fact that AD infrequently is not indicated in separation. It coexists with vascular pathology, demanding diagnostic works that consider mixed etiologies (83).

Brain resorption typically disrupts an individual’s brain activity and affects many regions within the brain (84). The development of the brain is influenced, to some extent, by an individual’s genetics and a correlation exists between AD and a progressive reduction in brain volume or capacity which can be recognized by image processing. The genetic factor holds potential as a prominent diagnostic indicator for AD (85). The lack of exceptions in AD is contingent upon genetic factors rather than environmental factors (86). Most image processing techniques entail conceptualizing the image as a two-dimensional signal, followed by applying conventional signal processing techniques to manipulate and analyze it. Manual, or semi-manual, approaches to these modifications already exist (87). In short, many image processing techniques could assist in determining AD using simple image processing approaches, as the authors will mention below.

**FIGURE 8:**
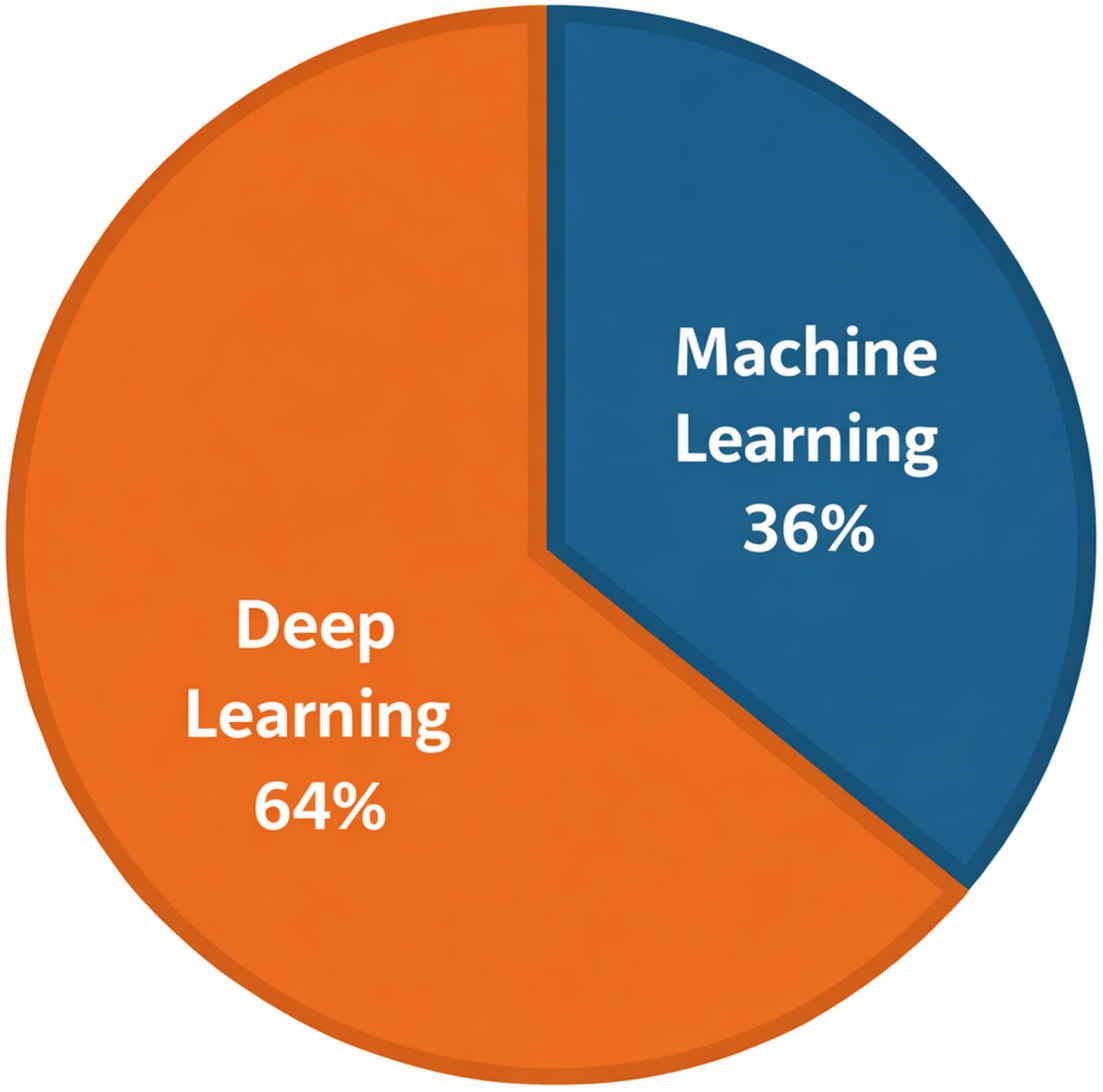
Output of applying Otsu thresholding in the brain.

In 2020, a study introduced a new automatic thresholding algorithm for gray images using image gradient information (88). Automatic thresholding is a vital image processing technique that extracts the target areas from the background based on gray surface information (89). Many methods have been proposed to provide a suitable threshold value for an image with a gray surface histogram. However, most of these methods cannot accurately and efficiently divide the image by distributing the gray surface without error (90). A study also proposes a novel approach to determining the optimal threshold to calculate the amount of gray gradient per pixel through multiplying the element by image blot and Sobel masks (91). By applying Sobel edge detection in the continuation of the gray slope distribution, based on the statistical average results, the average gray slope values of the pixels with different gray surfaces are obtained (92). Thresholding and edge detection methods such as mentioned above are used to segment brain regions in order to enhance biomarkers or “features” extraction for AI approaches training. Figure 8 illustrates the various outputs examined for analyzing medical images using Otsu and artificial neural networks. The provided images can be categorized into four rows. The first row contains brain MRI samples. The second displays a series of noisy images. The third and fourth rows exhibit binary and threshold images, respectively. The method employed demonstrates a partial capacity for detecting brain tumors. The utilization of Otsu thresholding in the diagnosis of diseases, specifically in the extraction of brain tumor edges and borders (as this thresholding’s main purpose is to find the region borders based on pixel intensities) has significantly enhanced the likelihood of successful outcomes for patients (93).

A narrative review paper published in 2025 documented a novel instance of AD factors being reported at a frequency of one occurrence every three seconds (94). Dementia is a condition that impacts a global population of approximately 50 million individuals, with projections indicating a significant rise to around 152 million individuals by 2050 (95). According to a study conducted in 2021, the projected expenditure for dementia treatment was $1 trillion (96). There is a notable disparity in the rate of increase of dementia cases between low- and middle-salary countries and high-salary countries.

The assessment, diagnosis, and treatment of AD in new patients lacks practical and conclusive universal approach (97). Early detection, however, is highly effective in mitigating the impact of the illness and maintaining well-being (98).

**FIGURE 9:**
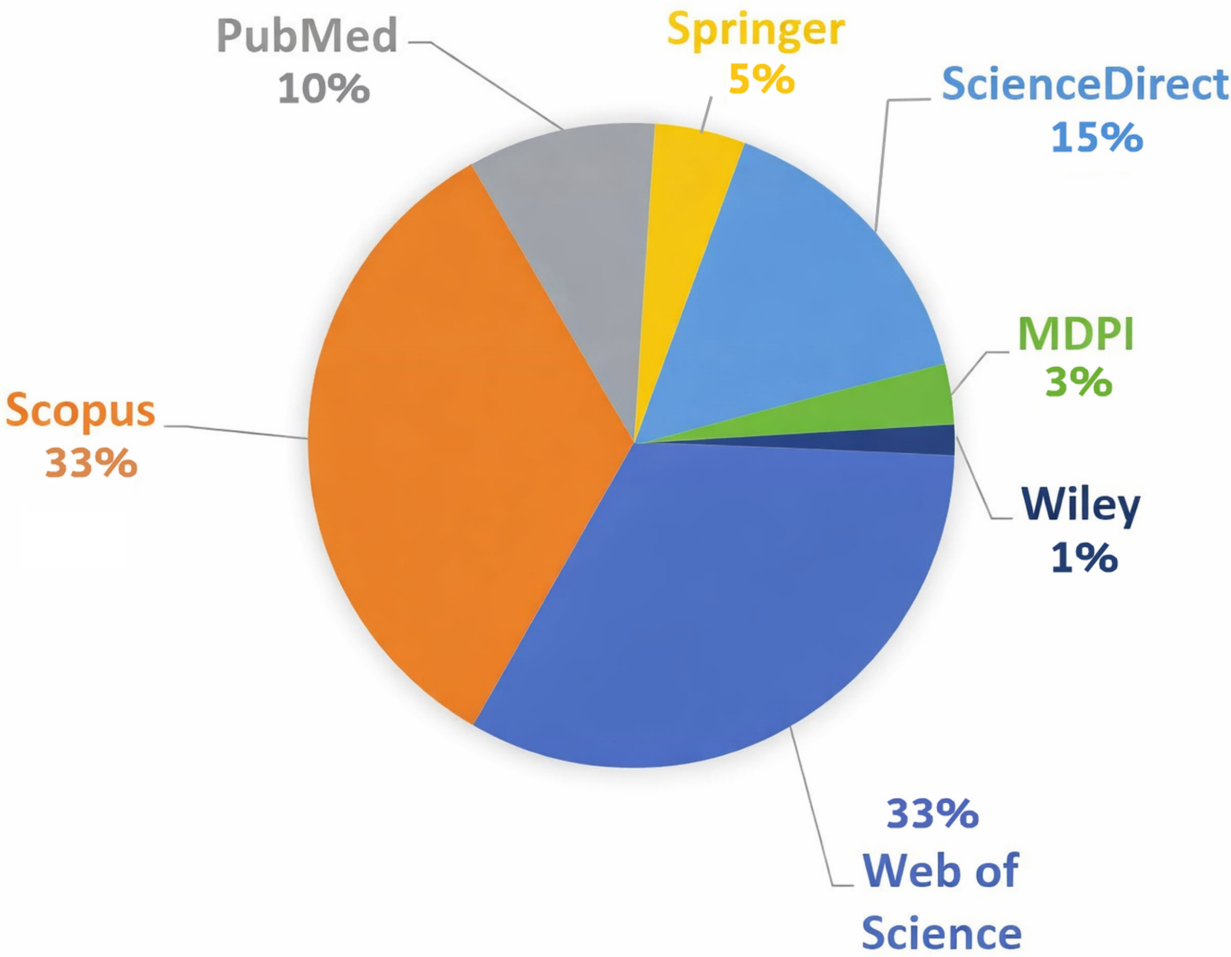
Regions impacted by the disease in the brain.

**FIGURE 10:**
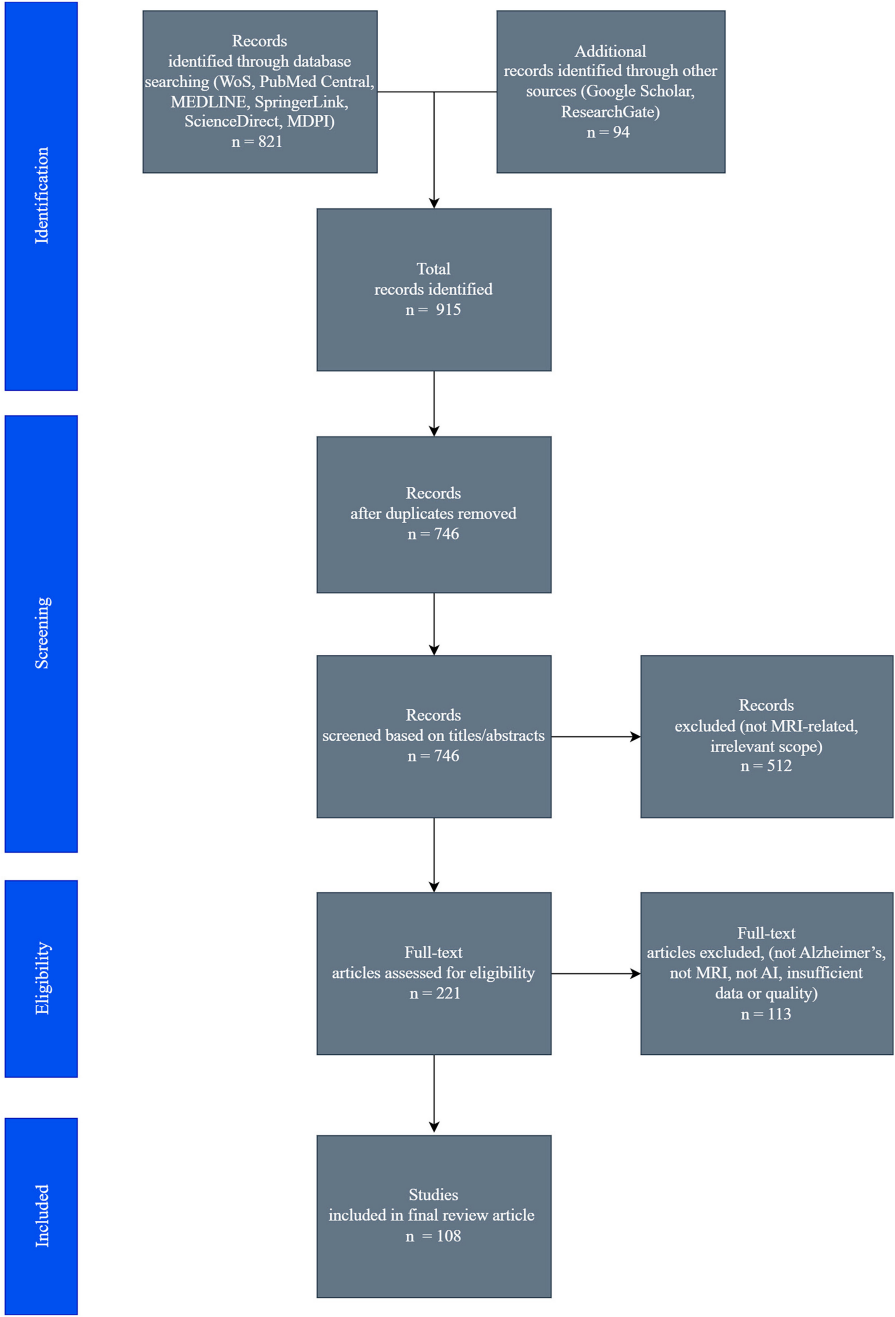
MRI real scans for healthy and unhealthy brain.

AD is influenced by many factors, including lifestyle choices, cardiovascular disease, cranial or cerebral surgical procedures, advancing age, gender, genetic predisposition, infections, environmental influences, and underlying medical conditions such as diabetes. In the context of AD, it has been observed that spherical protein structures are present in specific brain regions, located outside the neurons (99). Additionally, stringy protein structures have been identified within the cell bodies of neurons. Amyloid plaques are spherical protein structures formed by AD, which is characterized by brain tissue damage and the deformation of neuronal components (100). Figure 9 (101) and Figure 10 (102) present the adapted structure of brain tissue associated from AD which can be utilized by AI models.

### 4.2 Machine learning (ML)

Various techniques are utilized for disease diagnosis through the analysis of these images and the use of ML algorithms, including supervised and unsupervised approaches. One of the proposed methodologies involves a two-step process (103). The initial stage involved the Contour-based Brain Segmentation Method (CBSM) to eliminate the skull from the image data. Subsequently, the Quick Fuzzy clustering technique was employed to segment the brain tissue, specifically White Matter (WM) and Gray Matter (GM). In the subsequent stage, the analysis involved the computation of similarity measures, such as the Jaccard and Dice coefficients, between the normal brain and the segmented WM and GM. This analysis aimed to identify the presence of AD in Magnetic Resonance (MR) brain images (104). Another work in literature utilized an SVM ensemble method that merged bagging strategy without replacement or additional possibilities. In case of multi-variate classification of dementia, the SVM was well known to be the most applied approach when creating the AI model. Consequently, it proved beneficial in assessing the potential efficacy of this ensemble of classifiers. The objective of the studies was to hire a Support Vector Machine (SVM) model to diagnose AD using magnetic resonance imaging of the brain (105).

The study from Akbar et al., examined four distinct optimization algorithms, namely the Genetic Algorithm (GA), Particle Swarm Optimization Algorithm (PSO), Gray Wolf Optimization (GWO), and Cuckoo Search (CS), in the context of diagnosing attention deficit disorder (ADD) using brain sub-regions. Among the various optimization methods, the GWO showed promising outcomes in obtaining the optimal global solution. The segmented regions were classified using a ML classifier and evaluated using Ground Truth (GT) images (106). Other authors pre-processed MRI scans of the brain using an adaptive histogram. They decomposed it into four IMFS using Bidirectional Empirical Mode Decomposition (BEMD) for this analysis, and a Computational Intelligence (CIT) tool for AD diagnosis. Local Binary (LBP) patterns are then measured and concatenated with IMF histograms (107).

Subsequently, a recently proposed and enhanced Residual Network (ResNet) and an Advanced Computational Neuroscience Network (ACNN) have been implemented. The diagnostic accuracy of the two models was evaluated by performing inference or checking using an additional 469 distinct sets of 3D MRI images. Compared to the CNN network, the new ResNet exhibits a superior performance. This is evidenced by its statistically significant improvement in accuracy and its reduced occurrence of fulfilling positive outcomes (108). Table 2 presents an overview of the various methodologies commonly employed in investigating the detection of AD through MRI image analysis. The standard datasets used in the literature are Open Access Sequence of Image Studies (OASIS), which consists of structural MRI data, and Alzheimer’s Disease Neuroimaging Initiative (ANDI), which records data research aimed at improving clinical trials for the disease.

**TABLE 2:** Various ML models in the detection of Alzheimer’s.

Reference	Experiment data	Methodology	ML type	Average accuracy (%)	Limitations
Sadegh-Zadeh et al. (105)	Tehran University of Medical Sciences	Various ML classifiers	Supervised	89	No Severity, Random Forest and AdaBoost, and Extra trees have almost the same accuracy, shortage of data due to specific clinical scores
Vanaja et al. (196)	ADNI + OASIS	Integrative approach combining VBM	Hybrid	88	Computationally intensive, multimodal fusion complexity
Suk et al. (197)	ADNI MRI	SVM	Multimodal fusion	91	Requires multimodal data, PET scans not always available
Li et al. (104)	ADNI	Fuzzy-based system	Both	83.4	likelihood hypothesis, accurate outcomes, shortage of data
Sørensen and Nielsen (136)	ADNI	Ensemble SVM	Supervised	55.6	Data Scarcity, SVM computations
Clark et al. (177)	UCLA and ADRC	Ensemble of classifiers with four different architectures: random forests of conditional trees, support vector machines, naive Bayes, and multilayer perceptions	Supervised	84.1	Simple model complexity and Data Scarcity, some decisions in trees
Salami et al. (110)	Open Access Series of Imaging Studies (OASIS-3)	Clinical Decision Support System (CDSS)	Supervised	90.1	OASIS-3 data
Ghazal et al. (142)	MRI dataset	Transfer learning on multi-class classification	both	91.70	Limited Epochs number, Dataset Scarcity
Baydargil et al. (143)	ADNI	GAN-based anomaly detection based on skip connections	Supervised	96.03	Skip connections, increase complexity, memory usage, and heavy tuning requirements

Sriram et al., proposed ensemble learning techniques on MRI data, showing improved accuracy in early Alzheimer’s detection compared to traditional ML models (109). The classification was based on the analysis of three-dimensional MRI images. In addition, other studies outline a method for categorizing three-dimensional data for 178 individuals, 97 without cognitive impairments, and 85 with mild cognitive impairment or AD (110).

Initially, it was necessary to pre-process all 3D images to achieve a standardized numerical range within the images. Subsequently, the 3D wavelet transform was employed to analyze and extract the wavelet coefficients. Later, the triple attributes of energy, variance, and Shannon entropy were derived from the sub bands of the wavelet transform. Principal Component Analysis (PCA) was employed to diminish the attributes (111). Next, the classification task was performed using SVM. Lastly, the DL approach was employed, and its performance was assessed by comparison to the sup-port vector classifier (112).

**FIGURE 11:**
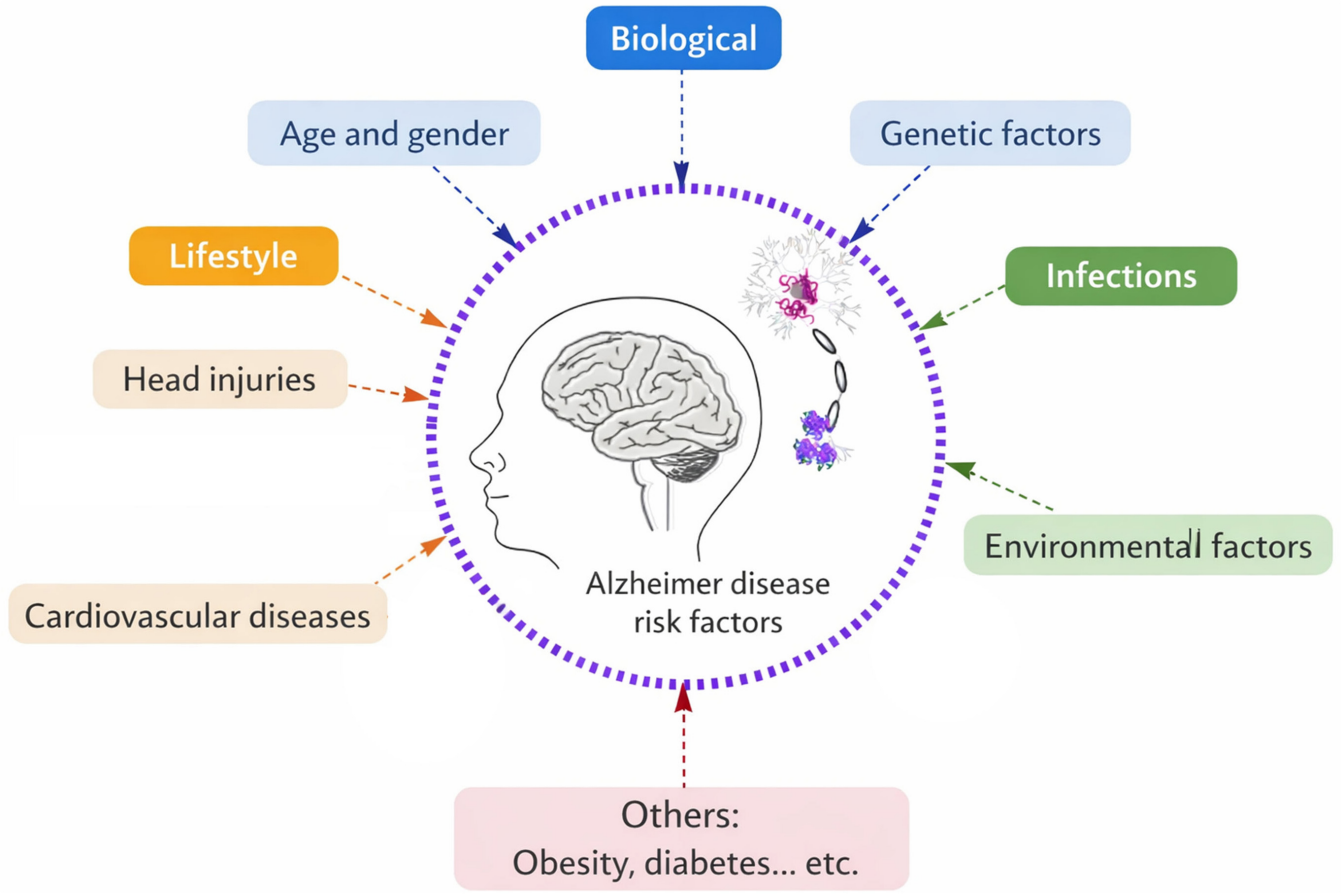
Use of color Intensity for the brain in AI models.

The study published by Ali et al., focuses specifically on the optimal feature selection as distinct brain tissues. By studying their MRI driven AD, they merged two methods using canonical correlation approach (113). The hypothesis was derived from the measured data and the observed intensity in that region as they claimed that this improved the classification performance as they got 94.7%. Figure 11 illustrates a visual depiction of a particular area of the cerebral cortex, which has been emphasized using coloration (114).

Bhandari et al., in 2020, proposed a novel approach for image division at multiple levels, utilizing the lightning search algorithm. This study suggests a novel image fusion-based threshold prototype segment and a multilevel color image zone. The Lightning Search Algorithm (LSA) was utilized to achieve this objective (115). The Otsu thresholding method can operate on color images using variances derived from 3D histogram diagrams (116). The accuracy of these methods is diminished. To address this challenge, energy is used to manipulate the textual data associated with an image and is commonly observed in numerous studies of the curved model (117). A study by (118) utilized transfer learning on MRI data for early detection of AD, they showed strong results in terms of generalization across heterogeneous cohorts.

Yet, utilizing the energy model concept created a high level of difficulty and proposes a lightning optimization algorithm in which it claimed as novel optimization that inspired by the characteristics of lightning phenomena, specifically the rapid and robust nature of storm lightning. The approach applied a lightning search optimization to identify thresholds for comprehensive investigations to be optimal. In addition, a fusion with local contrast is proposed in order to enhance the quality of the multi-level image segmentation. This scenario demonstrated that the lightning search optimization outperforms many existing methods in settling the optimal image thresholds through a comparative analysis of existing limitations (119).

A study conducted in 2020 proposed an efficient algorithm named shrimp algorithm, this algorithm addressed the color issues associated while partitioning threshold of a multi-stage image. Classic thresholding methods demonstrated good efficiency when applied to two-level thresholds, but the computational complexity resulted in multi-level thresholds of coloring can be significantly raised. Dividing a multi-level color image into thresholds can be considered a finite optimization problem. To overcome this challenge, a group of AI algorithms, commonly applied to over cross complexity, can be employed for adapting the results. These algorithms were the shrimp without any genetic operators, the shrimp algorithm with mutation, the shrimp algorithm with combination, the modified firefly algorithm (MFA), the modified locust optimization algorithm (MGOA), the bat algorithm (BA), and the water cycle algorithm (WCA). The purpose of creating such methods and comparison is to evaluate the effectiveness of the proposed model within these algorithms which are subject of research. The study involves conducting experiments on ten standard color images to determine the optimal threshold values, target values, PSNR index, SSIM, and standard deviation from target values at various levels. As a result of the experimental scenarios, the shrimp algorithm partitioned a color image’s multi-level thresholds which showed to be more effectively than the other algorithms even those thresholding approaches can be used also to identify AD brain effected regions (120).

When research applied to multi-level thresholding of color images, the Kapoor Entropy yields a superior accuracy rate, an automated MRI scan classification based on acquired texture features and gravitational search algorithms (121). Segmenting and classifying MRI brain images has emerged as a challenge issue for radiologists in modern era, in traditional brain image analysis methods, decision-making processes were inefficient and imprecise. To avoid these constraints, an assisted automated detection had been introduced to literally categorize the normal and abnormal spots in brain MRI scans. The model under consideration can assist radiologists to detect brain MR anomalies or irregular patterns at their earliest phase. Before segmentation, the histogram of images was adjusted with a limited contrast in order to enhance the quality. The region of interest was partitioned from other regions utilizing the Otsu multi-level threshold algorithm (122). Furthermore, a proposed system employs a selection process to identify the most significant and pertinent features from contextual and multimedia sources. Multi-solution properties are extracted using discrete wavelet transforms, fixed wavelet transforms, and fast discrete curve conversion techniques. Furthermore, the utilization of a binary and local pattern is employed to extract texture properties from images (123). The characteristics are used for categorizing brain MR images through a neural network classifier with a nutritional aspect. This classifier is improved by adding multiple meta-heuristic optimization algorithms, including the genetic algorithm, particle optimization, and the gravitational search algorithm, the error reduction technique was performed (124).

The task of ML classification involves determining the membership of a novel observation within a predefined set of categories or sub-populations. This determination is made by utilizing a set of features derived from training data, which is employed to train the classification model (125).

Supervised learning constitutes a distinct subset within the broader field of ma-chine learning. The approach described is a commonly employed technique in ML. It involves presenting a system with a collection of input-output pairs to enable the system to acquire knowledge and develop the ability to generate output based on a given input. Supervised learning necessitates the presence of input data to train the system. Some examples of supervised classifiers include support vector machines, boosting algorithms, nearest neighbor classifiers, and artificial neural networks. The utilization of Support Vector Machines (SVM) was employed as a methodology in these studies (126).

A review study conducted in 2025 showed a ML approach to classify and diagnose AD by analyzing retinal arteries (127). Their research findings indicate that always if the authors could early treat through regular screening would enable patients to control the long-term consequences of this disease. At the same time, the existing clinical diagnostic imaging tools are characterized by their high cost (such as MRI) and restricted capabilities (such as CT). This study proposed an innovative approach for diagnosing AD patients by utilizing retinal assessments, explicitly focusing on retinal arteries, as a new model to overcome traditional screening methods. The authors showed patients’ retaining image data as input in this article. Another study employed support vector techniques to classify the images into two categories: healthy individuals and those diagnosed with AD. The authors utilized the UK Bio-bank dataset for their evaluation and achieved an average classification accuracy rate of approximately 82.44%. The analysis revealed that within retinal images, the presence of small vessels yields a more significant amount of diagnostic information about AD. One benefit of creating this model is the usage of novel ways and data sources, such as retinal images, to generalize the rates of diagnosing AD. However, a significant obstacle that needs to be addressed is the comparatively accuracy of this method in effectively diagnosing AD with other proposed aspects (128).

A study evaluated segmentation techniques towards diagnostic tumors in the BraTs 2018 data set on three-dimensional MRI images with T2 weight image (T2WI), a primary pulse sequence used in MRI [127]. The process of sequence weighting underlines variations in the T2 relaxation duration in tissues. The techniques evaluated include pixel linear binary pattern, area growth, and Otsu threshold to compare similar efficiencies to the proposed method. In this study, three-dimensional MRI images are first exposed to incisions, which are about 10% of the top and bottom of the standard image, as these incisions do not have significant detail and strengthen the tumor areas on regular incisions. It uses amplified kernel processing in various experiments with different brain core sizes. Enlarged tumor areas are exposed to the local thresholding technique to extract the tumor from regular incisions using the kernel concept. Detected tumors are compared with the results of segmentation and manual zoning to determine the effectiveness of the proposed method. The results show that the proposed method has an average Jacquard similarity of 89.18% (129).

### 4.3 Deep learning (DL)

One of the feasible approaches for the diagnosis of AD involves the utilization of DL and ML techniques. A lot of these techniques are utilized for image classification, specifically in classifying images or their respective regions (130).

Table 3 presents an overview of the CNN with Deep layers that have been widely employed, their respective parameters, and the energy consumption related to the architecture applied. The “low/moderate/high” parameters are calculated based on the processing time and energy required to achieve a specific computation in the same hardware specifications. For instance, in the GoogLeNet model, the number of parameters generated by its kernels takes 10 times longer to be processed than the number of parameters generated by LeNet model (131).

**TABLE 3:** Overview of the main CNNs with deep layers.

CNN model	Number of layers	Number of parameters	Complexity and energy consumption
LeNet	5	60,000	Low
VGG-16	16	138 million	Moderate
AlexNet	5+3 Fully Connected	62 million	Low
ResNet-18	18	11.5 million	Low
GoogLeNet	27	62.3 million	High
DenseNet-121	121	7.6 million	High
R-CNN	3	72,960	Moderate

In long short-term memory (LSTM), a version of recurrent neural networks (RNN), The LSTM unit contains an input modulation gate, an input gate, a forget gate, and an output gate, each corresponding to four distinct sets of parameters. The total number of parameters of RNN = input vector × number of units + bias (number of units) × number of units (recurrent connections) (132).

In addition, Vision Transformers (ViT) appeared recently as a powerful alternative to CNN, which now represents the state-of-the-art (SOTA) in several image identification tasks within computer vision. ViT models surpass the existing state-of-the-art CNNs by fourfold in computing efficiency and accuracy. Transformer models have established themselves as the prevailing standard in Natural Language Processing (NLP). The widely used ChatGPT AI chatbot is a transformer-based linguistic model. It is specifically founded on the GPT (Generative Pre-trained Transformer) architecture. This employs self-attention processes to represent the interdependence among words in a text. Recent interest in computer vision research has surged around Vision Transformers (ViTs) and Multilayer Perceptron (MLP) (133).

The study carried out by Jain et al., in 2019 proposed a method using Positron Emission Tomography (PET), a scientific method used to assess physiological functions by examining blood flow, metabolism, neurotransmitters, and radiolabeled medicines. PET provides the capability for quantitative analysis, enabling the monitoring of relative changes over time as a disease progresses. The authors in this paper used PET images with a Generative Adversarial Network (GAN) DL model. The accuracy rates for diagnosing AD patients have approximately reached 96.03%. One benefit of employing this approach lies in its use of DL network informed in game theory, which results in an excellent level of precision. This approach is instructed by utilizing a limited quantity of training samples. As a challenge, this approach considered that it took a lot of time required for DL training in GAN networks (134).

Sørensen and Nielsen presented a DL-based classification approach for identifying AD plaques in MRI scans with a sensitivity rate of 98% which is excellent. However, it is important to know that this method’s accuracy is limited to a specific set of images, posing a challenge in general aspects. Another previous study suggested by the same authors used DL techniques to diagnose AD based on MRI images. The study employed VGG19 and DenseNet169 architectures as the DL architectures (135). The experiments conducted that the model possess an interesting capacity for patient classification. Still, an urgent challenge associated with this method existed which is the absence of regionalization in order to separate the regions effectively (136).

The study’s authors introduced a DL approach in their work, as documented to make it easier for the diagnosis of AD through the analysis of MRI scans. The findings from their experiments indicate that the ResNet architecture, when implemented with a huge number of layers, yields the most favorable outcomes concerning the diagnosis of AD. Yet, a primary challenge encountered in this article is the absence of a robust feature selection phase in their proposed approach (137).

An article analyzed 6735 brain MRI images to automatically classify and diagnose AD. The study examines existing literature, which had proposed a suitable approach for the automated diagnosis of AD using magnetic resonance imaging (138). Another study introduced an ML approach for classifying and diagnosing AD utilizing retinal vessels. The mean classification accuracy observed was reached 82.44%. The benefit of employing this approach is its utilization of novel models and recent and new datasets, including retinal images, to diagnose AD. The main limitation of this approach lies in its comparatively reduction of precision rates when employed to diagnose AD (128).

A study developed a hybrid deep learning model integrating VGG16, MobileNet, and InceptionResNetV2 for precise identification of AD markers from MRI scans (139). The research findings of a comprehensive literature review of DL in diagnosing AD indicate that, while DL had achieved good improvements in diagnosing AD, these approaches are subject to several constraints, such as the quality and quantity of existing datasets and the substantial investments necessary to train those models. In our review, the authors focused on exploration of the limitations and complexity of each model in relation to energy consumption (140). An analysis of the time series data was presented in a study created by Donnelly-Kehoe et al., to detect the progression of AD.

Ben Ammar and Y. Ben Ayed proposed a classification approach involving binary and multi-class classification tasks by utilizing 3D convolutional neural networks. They compared three different data augmentation methods on the overall effectiveness of CNN architecture in the three-dimensional field, specifically for detecting AD at an early phase (141). The evaluation conducted by the researchers demonstrated that their proposed method exhibits superior accuracy when compared to traditional CNN architecture. The study in 2020 introduced a new model for extracting brain tumors from three-dimensional MRI images using core processing and adaptive thresholds (142).

The analysis of anomalies and AD in PET images was proposed in a study conducted in 2021. The researchers employed an unsupervised deep-learning model to perform this analysis. The study aimed to examine the aberrations associated with AD by analyzing PET images, employing a novel unattended hostile model. The present model is comprised of three distinct components. The parallel network encoder is made up of two parts: a convolution pipeline and an advanced convolution pipeline. The convolution pipeline extracts features, while the advanced one combines these features (143). During the preprocessing stage, the input image is reconstructed by a receiver using the feature vector and a separator that distinguishes between genuine and fake input images, the latter being images generated through random noise distribution, so it should be taken care of when using any input to improve transparency (144).

An analysis has been proposed to classify AD, mild cognitive impairment, and normal control groups into three distinct categories. The evaluation conducted demonstrates that the proposed model achieves an average accuracy rate of two cohorts with 86% in diagnosing individuals with AD (145). One feature of an approach conducted in 2024 that makes it commendable is its use of a DL network based on imbalanced classification. This method also imparts instruction with only a few or no training examples. However, GAN networks require considerable time for DL, which can be a significant challenge (146).

A novel approach utilizing DL to diagnose AD plaques through image classification was introduced in 2021. In this study, the authors developed a DL model for image classification that discriminates between astrocytes and neurite plaques. The classification was based on analyzing tissue sections treated with phospho-tau immunohistochemistry (147).

This methodology uses an automated DL to construct a diagnostic model for detecting AD plaques. A dataset consisting of 1,500 files from 72 patients was utilized. From these files, 1,332 images were applied for educational purposes, while the remaining 168 were reserved for validating the DL techniques. The use of the cross-validation method has yielded evaluations indicating a sensitivity rate of approximately 98% for this approach. Furthermore, the accuracy of this method has been consistently reported as being exceptionally high. One notable benefit of this research lies in the high accuracy exhibited by the proposed method. However, it is essential to acknowledge that this method’s accuracy is limited to a specific set of images, which presents a challenge. One of the primary obstacles pertains to utilizing pre-existing DL models as their proposed approach, as well as the absence of originality in their research endeavors (148).

Zhou et al., proposed a methodology which is published in July 2025 for detecting AD early using MRI scans through DL 3D Convolutional Block Attention Module. According to their research findings, it has been observed that, on a global scale, an individual contracts the ailment, as mentioned earlier, at a frequency of once every four seconds. Furthermore, it has been established that this affliction, when it reaches an advanced stage, ends in death. This study presents DLbased model that utilizes MRI brain images as input to classify individuals concerning the presence or absence of AD. The experimental results demonstrate the significant capability of this model in patient classification. However, a major limitation of this approach is the extended training time (149).

Subramoniam et al., introduced a comprehensive machine-learning approach in 2021 to facilitate the diagnosis of AD through the utilization of magnetic resonance imaging. This study introduces DL approach that uses a self-coding deep neural network framework in conjunction with magnetic resonance imaging. The findings of their experiments demonstrate that state-of-the-art image classification networks, such as VGG and ResNet DL networks, exhibit encouraging outcomes in diagnosing AD. The research and experimentation conducted by the authors demonstrate that the use of transitional learning enhances the efficacy of DL algorithms in diagnosing AD through magnetic resonance imaging of the brain. An analysis of the experiments conducted reveals that utilizing ResNet-based DL architecture, characterized by a considerable number of layers, yields the most favorable outcomes in diagnosing AD. This study’s primary obstacle is the absence of an efficient feature selection phase in their proposed methodology (150).

In a study published in 2021, brain MRI images and DL techniques were evaluated for their potential to classify and diagnose AD automatically. By examining the existing literature, this study aims to identify a suitable and precise method for the automated diagnosis of AD. Research articles about AD published in esteemed academic journals between 2017 and 2020 were comprehensively analyzed by this study. This review examines different approaches associated with modern tools used in early detection. To facilitate researchers’ understanding of current AD diagnostic algorithms and techniques, the study aims to provide a guideline (107).

According to the authors in a study conducted in 2021, structural magnetic resonance imaging was used to categorize AD. Such datasets as the ADNI can be used to classify individuals with AD using appropriate feature selection techniques that aim to identify smaller brain regions with the most significant features. Based on DL, SegNet is used in this article which is considered a novel approach to classification problems. Using MRI structural features and brain segments, the proposed method identifies patients and dementia conditions with high accuracy. A trained ResNet-101 model was used. The 240 structural MRI images were analyzed to extract seven morphological features including gray matter, white matter, cortical surface, gyrus and sulcus contours, cortical thickness, and hippocampus. For training ResNet, the SegNet learning model was used. In the experiment, the classification method was found to be 96% sensitive and 95% accurate. Using this approach minimizes the input dimensions for learning with feature selection techniques. A significant challenge associated with this method is the considerable amount of time needed to train and classify samples (151).

A further comprehensive overview of DL methodologies used in AD diagnosis was published in 2020. The review indicated that AD is a major cause of mortality in developed countries. It documented that DL algorithms can be used to diagnose AD from a research standpoint. Medical imaging analysis and interpretation have greatly benefited from the widespread implementation of deep models in recent years. Since 2013, increasing emphasis has been placed on applying in-depth learning techniques to diagnose AD. Over the past few years, there has been a rapid increase in the publication of scholarly articles in this area. The researchers found that specialized models are more accurate in detecting AD than generic machine-learning models. It comprehensively explores the current condition of AD diagnosis. An analysis of over 100 scholarly articles on the diagnosis of AD was conducted for this study. It also examined how the brain works. An in-depth analysis of various models and an evaluation of their performance are provided in this study. There are some limitations to these approaches, even though extensive research into AD has yielded impressive results. Data acquisition challenges and the investment in time required to train these methods are among these limitations (152).

Using time series data, a study developed a multimodal DL model to diagnose AD. Timely diagnosis is very important for such a disease as it slows down the advance of AD and mitigates its effects. This study presented a robust DL model that uses convolutional neural networks and a long-term, short-term memory network. Five different types of multimodal time series data are combined, along with supplementary background knowledge in the proposed multitasking model, to predict multiple variables simultaneously. To extract the distinctive features of the two approaches, the proposed model used a deep convolutional learning network and a two-way long-term, short-term memory network. With the present process, local properties are extracted from the background data using a feed neural network. As part of this method, optimal features were used to discern prevalent patterns when classifying and regressing. A dataset of 1,536 images was used to validate the model with five diagnostic methods for detecting AD. Based on the findings of their study, the proposed approach can accurately forecast the progression of AD in individuals (153)

Using deep canonical neural networks to classify AD patients into multiple classes was the primary recommendation (154). Researchers faced a significant challenge in identifying early-stage AD. To accomplish this objective, the present study explores approaches that leverage deep convolutional neural networks and ML to solve the problem at hand. MRI is increasingly used as a diagnostic tool in clinical trials for AD.

Table 4 presents an overview of the DL methodologies commonly employed in investigating Alzheimer’s disease detection through MRI image analysis and their limitations. The limitations mentioned in Table 3 affect the results of the provided methodology. Sometimes, these limitations can be considered as challenging stages during the implementation of the methodology, as it does not always fit the case and nor can it be generalized to all AI application problems as a perfect strategy. In addition, the results are also affected by the way data are utilized and how the authors processed the data, even if they used the same data. Moreover, using the same data does not mean getting the same results, as sometimes preprocessing, optimization, feature appropriate selection, and a lot of other factors will also affect the final outcomes.

**TABLE 4:** Some DL methodologies in Alzheimer’s disease detection.

References	Experiment Data	Methodology	Average accuracy (%)	Limitations/challenges
Jain et al. (134)	ANDI	CNN VGG16	95.37	Skipping preprocessing, No finetuning
Chitradevi and Prabha (135)	Ground Truth (GT) images	Hippocampus (HC) Segmentation, population-based optimization, and Deep learning	95	Feature Selections and Data Scarcity
Bian (178)	ADNI	Proposed ResNet network	85.07	Data Scarcity and Model Complexity
Liu et al. (198)	White Matter (WM) and Gray Matter (GM)	Multi-scale convolutional neural network (MSCNet)	96.86	Overfitting possibilities, Data Scarcity
Ebrahimi-Ghahnavieh et al. (199)	ADNI	LSTM	84.38	Classify AD subjects from healthy older adults only based on MRI scans
Liu et al. (200)	OASIS	CNN	91.40	Generalization performance is mentioned without investigation, and Dataset Scarcity
Koga et al. (147)	JNEN	Google autoML	98.3	Diagnosis not provided
Zhou et al. (149)	3D MRI image	3D CNN and Swin transformer	92.92	Training took very long time (500 epochs of training)
Buvaneswari and Gayathri (151)	ADNI	ResNet	95	Only supplying ROI images better than supplying raw images
El-Sappagh et al. (153)	ADNI	Stacked CNN and BiLSTM	92.62	Critical modalities in their study because of the lack of data
Mehmood et al. (154)	OASIS	VGG-16	99.05	Normalization includes a chance to remove significant features that are regarded as irrelevant or unwanted
Kumar and Agarwal (201)	Kaggle AD	ViT and DL	97.65	planar features from axial slices and utilizes weighted fusions that are not always enough to be generalized to other applications
Sorour et al. (202)	Brain magnetic resonance imaging (MRI)	CNN, CNNs-LSTM, CNNs-SVM, and Transfer learning using VGG16-SVM	99.92	Data Augmentation is necessary to obtain optimal outcomes
Ul Rehman et al. (203)	ADNI	ViT	96.8	Transfer learning assists in reducing the issue of insufficient training data. However, data size continues to affect model performance

**FIGURE 12:**
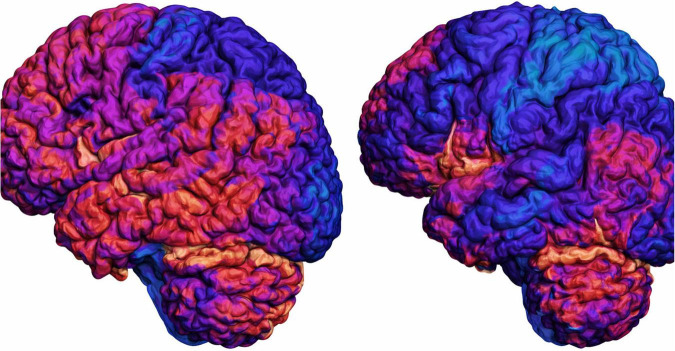
Right [ML and DL amount of data used for AI model (X-axis), Performance accuracy percentage (Y-axis)], Left [Amount of data with respect to AI Model Complexity of processing for AI model (Blue) and AI model Performance (Red) with respect to Complexity (X-Axis) and performance accuracy percentage (Y-axis)].

A study involved the development of a Siamese convolutional neural network model, which drew inspiration from the k-dimensional embeddings learning model to classify distinct stages of dementia. The proposed method was employed to conduct experiments on the publicly accessible ADNI and OASIS dataset, resulting in a diagnosis accuracy exceeding 91.83% for most samples concerning AD patients (155). Figure 12 shows the main difference between the ML and DL approaches regarding the complexity and amount of required data to acquire higher precision and better recall results.

ML and DL techniques show a lot of high potential. However, they continue to many challenges such as overfitting, which is still a major concern since a lot of models are trained on small, unbalanced datasets like ADNI or OASIS. These concerns made them less effective in other scenarios or data. Interpretability is another drawback, physicians often don’t prefer to trust black-box predictions even though they come with clear clarifications (156). Besides, inefficiency and high energy requirements restrict the ability to scale such as for large CNNs and transformer-based models. Insufficient generalization across different categories or patterns makes clinical reliability even worse, since AI models usually trained on standard data often do not perform well when utilized with real or real time hospitalization datasets (157). To find a solution for these challenges, researchers need to use explainable AI frameworks, energy-efficient architectures, and a wide range of multimodal datasets.

The main idea illustrated in Figure 11 refers to the ability of ML approaches to acquire knowledge from training data, which are the input data provided to the system. The extent to which these approaches can be learned is dependent upon the quantity of data available, and as the amount of data increases, there is no significant improvement in terms of achieving higher accuracy outcomes. On the other hand, DL methods can improve their performance as the amount of data increases, leading to increasing levels of accuracy. The model’s complexity has an immediate influence on its energy consumption and its overall complexity. However, it is important to note that a more complicated model does not necessarily guarantee a higher performance. It can occasionally have a negative effect and result in a loss of hardware efficiency.

### 4.4 Ensemble methods

The ensemble approach is a method used in ML that mixes multiple base models to generate a single optimal predictive model. Ensemble methods typically yield more accurate solutions than those caused by a single model (158). A review study created by (159) highlighted functional MRI techniques (ASL, resting-state BOLD-fMRI) combined with entropy-based analysis for differential diagnosis of AD. A study by Sone et al., represents one of the first attempts to utilize ensemble techniques in a higher dimensional space, specifically by mapping single nucleotide variants (SNVs) to genomic locations on bands (160). The integration of multiple models through this approach yields substantial in-formation, following the analysis of genome-wide association studies (GWAS) and random forest (RF) analysis. The novel Ensemble scoring algorithm is proposed to compute relevant information about bands that accentuate variations associated with diseases for datasets with varying attributes (161). The novelty of this scoring algorithm lies in its utilization of multiple data mining methods to extract knowledge about AD from each dataset. Utilizing the results from multiplatform prediction models as prior knowledge facilitates the integration of diverse information sources, leading to the development of a posterior ensemble model. This model employs minimization techniques to identify complex genetic variations associated with AD (162).

### 4.5 Entropy methods

Entropy, in the context of ML, quantifies the degree of randomness or lack of information in a specific dataset or system. Information entropy is a quantitative measure of the amount of information included in a dataset. It is frequently employed to assess the effectiveness of a model and its capacity to generate precise predictions (163).

The Shannon entropy is a mathematical function that quantifies the level of un-certainty associated with random variables (164). Methods utilizing the Shannon entropy are employed to assess the efficacy of predicting an event by considering the variables involved. Interactions between variables occur when the collective predictive capacity of a combination of variables in explaining an event surpasses the cumulative predictive capabilities of the individual variables (165).

Heinrich et al., introduced a novel method that utilizes mutual information to measure information gain. Additionally, they employed an interaction-information gain approach to analyze three-way interactions (166). The entropy-based three-way inter-action information method (3WII) is used to investigate the prioritized single nucleotide polymorphisms (SNPs) in each dataset, namely ADNI, GenADA, and NCRAD. Two-way mutual information and three-way interaction information are entropy-based methodologies utilized to quantify the level of interaction between two markers and the shared information among all three attributes (167).

In a case-control study design, the disease status of an individual is denoted by D = 0 for healthy individuals and D = 1 for affected individuals. Information gain is a term used to describe the disparity between the mutual information observed in the affected population as shown in Equation 1 and that of the general population (168).


I⁢G⁢(X,Y\D)⁢I⁢(X,Y\D)-I⁢(X,Y) (1)

The concept of interaction information gain for markers X, Y, and Z is similarly defined Equation 2:


I⁢I⁢G⁢(X,Y,Z\D)⁢I⁢(X,Y,Z\D)-I⁢(X,Y,Z) (2)

The calculation of the test statistics for information gain (TIG) involves dividing the information gain (IG) or intrinsic information gain (IIG) by a normalization factor of variance (Ʌ). The test statistics from the analysis follow a central chi-square distribution with one degree of freedom when considering the null hypothesis that the markers are independent of the disease (169).

The test statistics for each dataset were computed by utilizing the interaction in-formation obtained for the prioritized single nucleotide polymorphisms (SNPs). To identify interactions shared among all three variants and that cannot be accounted for by two-way mutual information gain alone, they excluded triplets consisting of SNP combinations that exhibit significant two-way mutual information gain (170).

### 4.6 Biological networks and techniques for their comparison

Knowledge of biological components and their interconnections is essential for comprehending several biological processes. It is not only proteins that are present at specific times during cell differentiation, but also their essential interactions. The best way to depict this information is through a graph, also referred to as a network, which accurately reflects the entities and their relationships. The description of it as a “network” is widely used for grouping interconnected or interdependent components. One instance of a network within the field of biology is represented by protein-protein interaction networks (PPIs). Proteins are essential catalysts, structural elements, signaling molecules, and molecular machinery within biological tissues. Protein-protein interactions (PPIs) are of utmost importance in orchestrating cellular processes and serve as the basis for numerous signal transduction pathways and transcriptional regulatory networks within a cell (171).

The fundamental variables that impact graphs are the number of nodes (N) and the networks’ average edge degree (k). The network topology, often undisclosed in experimental data, governs the inherent nature of the influence. Hence, it is essential to note that making direct comparisons of graph measurements between empirical networks with varying numbers of nodes (N) or edges (k) can lead to inaccurate findings (172). Comparing networks poses a challenge due to the inherent difficulty in assessing them as comprehensive entities. Networks can be reached using global properties and summary statistics, including but not limited to network density, degree distribution, transitivity, average shortest path length, and other relevant measures. Comparative analysis of networks characterized by different measures has been conducted by researchers (173).

Knowledge graphs (KGs) are a valuable tool in data science, as they can effectively manage various data formats and extract relevant information (123). Biological entities can be represented using knowledge graphs. According to Doðan et al., CROssBAR Knowledge Graphs represent biological entities with nodes. The graph’s edges are also used to express relationships between these entities, both within and across types (174).

The survey of related works of AI highlights several research gaps in applying AI for AD diagnosis. First, most studies rely on limited public data such as ADNI and OASIS, which restrict generalizability and raise diversity issues across populations and imaging protocols. Second, there is a lack of standardized benchmark frameworks, making it difficult to compare all outcomes across different studies and their utilized methods. Third, explainability remains underdeveloped as many DL models operate as black boxes, limiting trustworthy and adoption in clinical field. Fourth, few studies focused on computational efficiency and sustainability even with energy consumption of DL architectures. Finally, insufficient exploration of federated learning and privacy-preserving approaches found which could enable collaborative research without patient confidentiality. By addressing these gaps, it will be important in AI-based studies to turn them into clinically reliable tools for early AD detection.

## 5 Discussion and challenges

In this section, the authors discuss the intricacies and constraints associated with energy consumption and power processing in each AD diagnostic model. OASIS was utilized as a widely used dataset among scholars to enhance the effectiveness of their methodologies. Their study combined three Dense Net designs, resulting in an average accuracy of 98.82%. Their approach could potentially be applied to other medical fields. To establish the progression of AD, the authors used a publicly available dataset, ADNI (175). Besides, the researchers employed an ensemble SVM algorithm in their study and achieved a performance rate of 55.6%. Their work had many obstacles in limited available data and the excessive computational effort required for SVM calculations. 95% performance was achieved using segmentation and DL (176). However, they overlooked the crucial aspects of feature selection and optimization and the insufficient amount of data. The study by Bian introduced a ResNet network that utilized ADNI data. The study attained an accuracy of 85.07%. However, the key constraints were the limited data availability and the model’s complexity. In the study referenced as Clark et al. (177), a combination of four classifiers, ranging from random forests to multilayer perceptron, was utilized, resulting in an accuracy of 84.1%. The decision made in trees with many features may result in a high recall but low precision (178). According to a study by Ebrahimighahnavieh, the ADNI dataset they used, and the LSTM technique achieved an outcome of 84.38% (152). They asserted that they identified and categorized AD artifacts in cognitively normal elderly individuals solely using MRI images. A small number of epochs and a lack of data availability may have impacted the model’s performance which led to its lower accuracy. Utilizing transfer learning in a multi-class classification task enabled this. The primary limitation can be clearly described as insufficient data samples, which continues to be a significant obstacle that restricts the system’s functionality in a broader range of conditions (179). Additionally, the optimal selection of the model with the most effective fine-tuning has created challenges in generalizing the model for other medical applications. The authors believe that they employed a normalization technique that eliminated crucial aspects and retained only a limited number of features, which resulted in these observations. The main challenges are associated with widely utilized DL and ML methodologies, their corresponding parameters, the energy consumption linked to the implemented architecture, and the size of the data, even in case of ViTs. The low, moderate, and high consumption are selected according to the processing time and energy necessary to perform a certain computation within the precise hardware constraints. For instance, in GoogLeNet, the number of parameters generated by its kernels requires ten times more processing than the number of parameters produced by the LeNet architecture. Occasionally, these constraints may be viewed as formidable phases in the execution of a methodology, as it is not always feasible to adapt or generalize all AI application issues into an optimal strategy.

Recent outcomes from AI related studies demonstrate the potential assistance and possible challenges that created while executing such scenarios from AI-supported methods that are used in AD diagnosis. For example, a paper from Dardouri (29) showed that Deep CNN relying on MRI scans achieved substantial accuracy in early identification of AD. However, it experienced issues with generalizing their findings of a variety of various datasets. Also, a paper from Christodoulou et al. (180) examined PET-MRI usage and showed the value of how integration of different methods of imaging could affect the reliability of medical diagnoses. A case study carried out by (181) provided an explainable DL framework for EEG-based assessment of Alzheimer’s and frontal-temporal dementia, thereby indicating transparency in model decisions may strengthen healthcare confidence. Besides, a paper from Nguyen et al. (182) conducted an exploratory review of explainable AI identification, highlighting interpreting ability as a significant weakness in existing studies. Finally, a research paper published in The Lancet verified a reliable AI-directed biological marker that can predict early dementia in different categories of individuals (183). It depicted the fact of how important it is to create extensive and multi validation tests before utilizing it in clinical environments.

Benchmarking correlations between AD and other neurodegenerative disorders is essential for clinical application (184). Genomic studies show that AD, PD, and vascular dementia all have similar ways that proteins fold wrong and cause oxidative stress (185). Imaging studies further validate that cerebrovascular disease serves as a mediator of cognitive decline in AD, with heterogeneous pathology commonly identified in clinical associations (186). AD datasets indicate that as many as 40% of patients exhibit concurrent vascular and neurodegenerative characteristics, thereby complicating diagnosis (187). To make sure that AI approaches are strong enough and could be used in many different situations, it requires testing against both pure AD situations and mixed pathology related diseases.

In comparison of ML and DL on common datasets such as ADNI and OASIS. In case of ML, SVM, Random Forest, kNN, logistic regression and even Ensemble methods are generally shown faster training, lower complexity or computational cost with less energy consumption as they work better with smaller datasets and biomarkers can be traced (188). The accuracy ranges between 82 and 91%. In case of DL such as VGG, DenseNet, ResNet, LSTM and ViT are consistently achieve excellent outcomes only when large MRI data are given or available as the accuracy performance improvement increases with more data. However, computational cost and its consequences such as energy consumption and training times are considered very high compared to ML algorithms. For interpretability, due to black box concepts of DL approaches, XAI methods are required (189).

Even though ML, DL, and Ensemble models show good results in terms of detection, they have some issues related to diagnosing AD. Traditional ML methodologies such as SVM, k-NN, and decision trees are considered limited due to the fact of the dependency on acquired features that don’t always capture the complex structural changes or abnormal behavior in MRI data. When trained these models on small datasets, these models also have overfitting problems which makes them less beneficial for all situations or cases. In DL models, such as CNNs and ResNet architectures, it solves the problems that could appear with feature acquisition, but they need good amount of data, variety in data and a lot of computing. Because they are considered black boxes, they are hard to comprehend, which makes clinical validation somehow difficult. Ensemble methods could make things better with more accuracy by combining several methods. However, they also could raise the complexity of the system, as they need more computing or high hardware configuration which can lead to duplicate feature selection. These limitations show that researchers need explainable AI frameworks, federated learning strategies, and standardized benchmark protocols to make sure that AI is used in such a way that could be beneficial futuristic in clinical settings.

AD is often correlated with other neurodegenerative and vascular ailments. Vascular dementia shares related features in case of AD, especially in white matter lesions and cerebrovascular impairment, which intensify cognitive decline. While Parkinson’s disease dementia and Lewy body dementia also exhibit structural and functional similarities, such as cortical and hippocampal involvement, making them relevant for comparative AI-based diagnostic studies. in addition, frontotemporal dementia caused by abnormal protein shows distinct atrophy patterns but could be misclassified as AD in early stages. In our case the authors are mentioning the AD cases. Enabling AI models to distinguish between overlapping anatomies among these ailments and improving clinical applicability.

Federated learning is a promising way to deal with the challenges of not having sufficient data and maintaining a medical imaging confidential. Federated learning allows organizations to train collaboratively without distributing raw patient data (190). This maintains sensitive MRI data to be locally saved while also creating enhanced and generalized models. These kinds of approaches that preserve privacy, such as differential privacy and secure retrieval, could assist in keeping patient data protected even further specially they are protected by patients’ acceptance to distribute their data (191). Recent AI architectures, for example AlzFed-XAI, indicate that federated learning can achieve accuracy comparable to that of traditional centralized learning while remaining easy to understand, which lets medical professionals understand how algorithms work (192). This strategy not only addresses ethical issues, but it also encourages collaboration on a large level between hospitals and research centers.

For the transition of AI approaches from research to clinical application, it is important to mention the need for standardized protocols. Benchmark datasets with standardized imaging recommendations or protocols should ensure that those AI approaches or clinical AI applications are trained, tested, and validated in the same way consistently (193). Fairness in terms of detection and evaluation across studies are possible with clear evaluation metrics. Repeatable workflows and standards of ethics that deal with bias, transparency, and safety for patients make reliability even greater. Without these types of standardizations, AI applications may remain for research only, and the experimental results might not be able to be repeated or reproduced at different institutions and clinicals (194). Recent agreement declarations highlight the demand for governments to collaborate rapidly to set international benchmarking standards for the usage of AI approaches in neuroimaging or any health-related studies (195).

Moreover, the outcomes are influenced by the data utilization methodology and the authors’ data processing techniques even when identical data sets are employed. Furthermore, utilizing identical data does not guarantee equivalent findings, such as preprocessing, optimization, appropriate feature selection, and several other factors can significantly influence the final conclusions.

## 6 Conclusion and recommendations

AI has made great strides in using data related to MRI or CT scans to diagnose AD. However, there are still many obstacles that keep it from being widely utilized in clinical or health organizations. Many current models or applications still use small or not very diverse data, which makes it more likely to have overfitting and less likely that they will work for a wide range of healthy cases/situations. In addition, some AI models also act like black boxes which makes it hard for doctors to have full understanding of what they mean or how they utilize it. Also, computational and energy requirements (hardware configurations) of DL architecture create sustainability issues, while inadequate clinical validation holds widespread confidence in these applications.

Three things required to be mentioned, the first one is that the usage of federated learning could help with data shortage by letting hospitals and research centers work together without having to share private patient data. This method protects privacy while allowing models to learn from bigger and more varied datasets. Second, researchers need XAI models to make predictions clearer and reliable. XAI helps doctors to be more confident and encourages responsible use in medical trials by explaining why a decision was made. Third, standardized benchmarking protocols must be done to facilitate fair comparison of AI tools in order to ensure reproducibility across studies and comply with regulatory protocols set by world health agencies such as the U.S. Food and Drug Administration (FDA), Medicines and Healthcare products Regulatory Agency (MHRA), and European Medicines Agency (EMA).

Future research should consider and concentrate on the integration of multimodal/data variety, which means merging other features such as blood tests, cerebrospinal fluid, and genetics. Hybrid and Fusion models can better understand the complexity of AD and help personalized medicine by making treatments fit each patient. The accuracy rates of AD diagnoses will improve even more with such scanners with higher Dots Per Inch (DPI) data. This will also make it possible to find neurodegenerative changes earlier.

AI models have made great progress in health devices and applications. One of these applications is the diagnosing of AD, but the field is still limited by a lack of data, issues with interpretation, hardware difficulties, and a lack of clinical validation. Researchers interested in AI approaches should not only focus on a bright scenario but also try to utilize methods that can be utilized in clinics by concentrating on federated learning, explainable AI, standardized protocols, and multimodal/data integration. This will assist physicians make rapid diagnoses, improve patient outcomes, and lessen the responsibility of dementia effect on society.
